# Microfluidics and fluorescence microscopy protocol to study the response of *C. elegans* to chemosensory stimuli

**DOI:** 10.1016/j.xpro.2023.102121

**Published:** 2023-02-14

**Authors:** Christine W. Bruggeman, Guus H. Haasnoot, Erwin J.G. Peterman

**Affiliations:** 1 LaserLaB and Department of Physics and Astronomy, Vrije Universiteit Amsterdam, 1081 HV Amsterdam, the Netherlands

**Keywords:** Biophysics, Cell Biology, Microscopy, Model Organisms

## Abstract

Here, we present a protocol to use microfluidics in combination with fluorescence microscopy to expose the *C. elegans* tail to chemosensory stimuli. We describe steps for the preparation of microfluidic chips and sample preparation through the sedation of *C. elegans*. We detail flow calibration and imaging of *C. elegans* through fluorescence microscopy to determine their molecular and/or cellular response to chemosensory stimuli. This protocol can also be applied to amphid neurons by inserting the worm in the chip head-first.

For complete details on the use and execution of this protocol, please refer to Bruggeman et al. (2022).^1^

## Highlights

• Preparation of microfluidic chips to expose *C. elegans* tail to chemosensory stimuli
• Sample preparation by sedating *C. elegans* and immobilizing a worm in the chip
• Calibration of the flows to ensure the worm is exposed to buffer or stimulus
• Fluorescence microscopy to study the molecular and/or cellular response in *C. elegans*

## Abstract (editor highlights)

Publisher’s note: Undertaking any experimental protocol requires adherence to local institutional guidelines for laboratory safety and ethics.

## Graphical abstract

**Figure:**
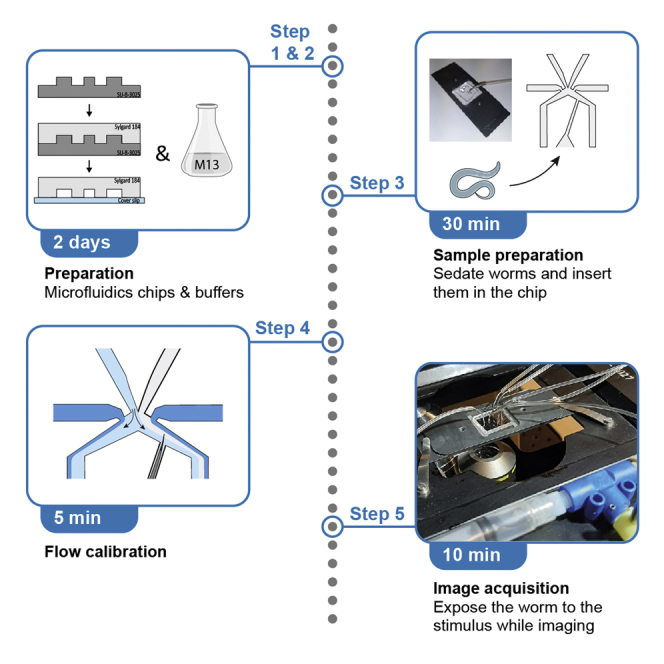

## Before you begin

The protocol below describes the specific steps for exposing the tail of *C. elegans* to chemosensory stimuli. It can also be used to expose the head of the worm, in which case the worm should be put in the microfluidics chip’s worm trap head-first.

For the protocol described here it is assumed that *C. elegans* strains are grown and maintained according to standard procedure, on nematode growth medium plates, seeded with HB101 *E. coli*, at 20°C. Other food sources, such as the commonly used OP50, are also suitable. Maintain your worm strains as required for your particular experiments.

### Preparation of microfluidics chips

**Timing: ∼1.5 days**

1. Create a master that can be repeatedly used to pour polydimethylsiloxane (PDMS) chips.
  a. The design of the microfluidics chip used in our study was identical to Chronis et al.^2^ (Figure 1A).

**Figure 1:**
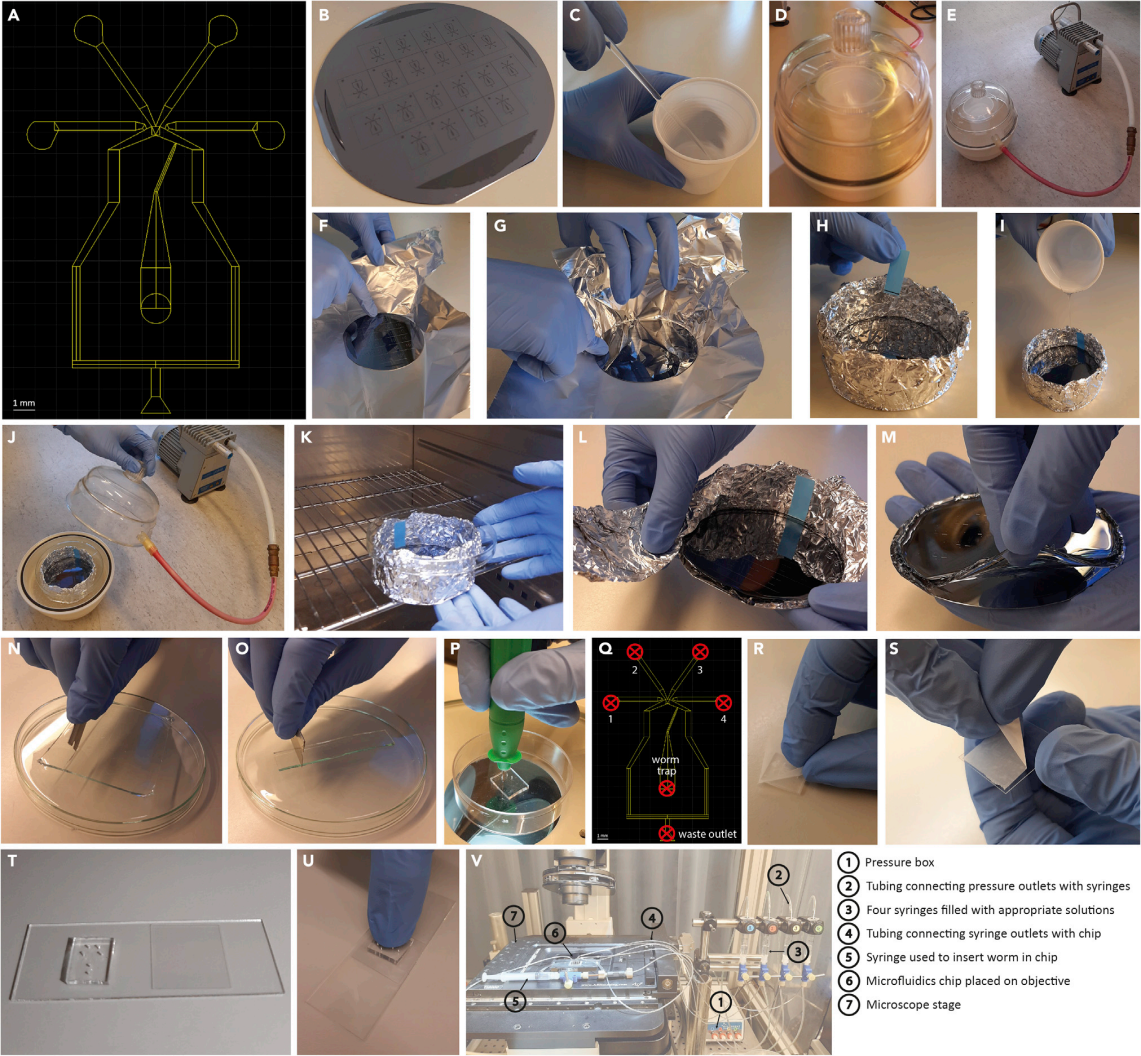
Preparation of microfluidics chips (A) Design of the microfluidics chip as reported by Chronis et al.^2^ Scale bar: 1 mm. The worm trap is 70 μm wide, 1,200 μm long and 28 μm high. (B) A master with the desired pattern of 20 individual chips, consisting of a 26–32 μm layer SU-8-3025 photoresist on a silicon wafer. (C) Mix the two components of PDMS thoroughly with a spatula. (D) Evacuate the PDMS mixture in a desiccator to remove air bubbles. (E) PDMS mixture in a desiccator. (F) Fold aluminum foil around the master. (G) Fold the aluminum foil in such a way that it becomes a ‘baking mold’. (H) Draw a line on sticky tape measuring 3 mm and put this inside the baking mold. (I) Pour the PDMS mixture onto the master. (J) Put the master with poured PDMS in the desiccator. (K) Cure the PDMS in the oven at 60°C. (L) Remove the aluminum foil. (M) Gently remove the PDMS from the master. (N) Cut the individual chips using a razor blade. (O) Cut the individual chips using a razor blade. (P) Punch holes in the appropriate places using a biopsy punch. (Q) Schematic overview of the microfluidics chip. The red crosses indicate the 6 places where holes should be punched. (R) Clean the surface of the chips from dust with use of sticky tape. (S) Clean the surface of cover slips with sticky tape. (T) Plasma clean the chips and cover slips. (U) Immobilize the chips on a glass slide. (V) Microfluidics set-up. Different parts are indicated by a number and explained on the right.
  b. A master with the desired pattern was created by AMOLF NanoLab Amsterdam, consisting of a 26–32 μm layer SU-8-3025 photoresist on a silicon wafer (Figure 1B).
2. Pour and bake PDMS chips.
  a. Mix the two components of PDMS (Sylgard 184, Dow Corning) in a ratio of 1:10, in a disposable plastic cup. Wear gloves when doing so, because it is difficult to wash the highly viscous PDMS components of your hands.
  b. Mix thoroughly with a spatula for at least 5 min (Figure 1C). Incomplete mixing will lead to soft spots in the final PDMS.
  c. Remove air bubbles form the mixture by placing the plastic cup, covered with a plastic lid (for example a petri dish), in a desiccator and evacuate for at least 30 min (Figures 1D and 1E).
  d. Fold aluminum foil around the master (Figure 1F) ensuring that the sides are folded a few centimeters up, as if it were a ‘baking mold’ (Figures 1F and 1G).
  e. Pour the PDMS mixture onto the master in the ‘baking mold’ (Figures 1H and 1I).
    ***Note:*** The less PDMS you add, the thinner the chips will be (this will reduce the autofluorescence when imaging), but be aware that you still have to insert steel pins for the fluidics. We prefer chips that are ∼3 mm thick. To know how much PDMS to pour, we draw a line on sticky tape measuring 3 mm, which we put on the inside of the ‘baking mold’ (Figure 1H).
  f. Put the master with poured PDMS, covered with a lid, in the desiccator and evacuate for an hour (Figure 1J).
  g. Cure the PDMS in the oven at 60°C, for 1 h (Figure 1K).
  h. Allow to harden overnight (>16 h).
3. Punch and seal the chips.
  a. Remove the aluminum foil (Figure 1L) and gently remove the PDMS from the master (Figure 1M).
  b. Cut the individual chips using a razor blade (Figures 1N and 1O). To make cutting go smoother, you can dip the razor blade in isopropanol.
  c. Punch holes in the appropriate places using a 0.75 mm diameter biopsy punch (World Precision Instruments) (Figure 1P).
    ***Note:*** The 6 locations (4 fluidic inlets, the worm trap and the waste outlet) where holes should be punched are indicated by red crosses in Figure 1Q.
    ***Note:*** In order to prevent damage to the puncher, use a plastic surface to work on (i.e., a Petri dish). See problem 1.
  d. Make the surface of the chips dust-free by using sticky tape (Figure 1R).
  e. Clean the surface of cover slips (22 × 22 mm) by using sticky tape (Figure 1S).
  f. Plasma clean the chips and the cover slips (Harrick Plasma, 29.6 W, 120 s) (Figure 1T).
    ***Note:*** If you notice later during sample preparation that the chips leak, you can increase the time of plasma cleaning. See problem 2.
  g. Immobilize the chips on a glass slide by gently pressing on it with your thumb for 10 s (Figure 1U).
  h. Cure the chips for 2 h at 80°C.
    ***Note:*** Pouring one batch of PDMS chips yields approximately 20 individual chips. As long as a chip is not leaking and the flows are properly set (See step-by-step method details), it can last for a few days of measurements. However, it can also happen that after one or several measurements, one of the channels gets blocked by PDMS or dust particles, in which case the chip can no longer be used.

In our experience, it works well to punch and seal chips in batches of 4. If one of the chips does not happen to work well, you still have a back-up to continue imaging.

### Preparation of buffers

**Timing: ∼4 h**

4. Prepare M13 buffer.
  a. See table in materials and equipment section for a detailed recipe.
  b. Sterilize by autoclaving, 20 min at 121°C, before use.
  c. Store at room temperature (18°C–25°C).

***Note:*** Use M13 and not M9, because M9 buffer causes precipitation of certain chemicals that you may want to use as stimulus, like CuSO_4_.

5. Prepare a 5 mM solution of levamisole (Sigma Aldrich) in M13 to sedate your worms.

***Note:*** For long-term storage, keep the levamisole at −20°C. Short-term storage (several days to about two weeks) can be at 4°C.

6. Prepare a solution of 5 μm fluorescein in M13. Store at room temperature (18°C–25°C).
7. Prepare solutions of the chemosensory stimuli you would like to test during your experiment, in M13. Examples of the stimuli used in this study: 0.1% SDS, 10 mM CuSO_4_, 0.5 M glycerol, 0.25 M NaCl. Store at room temperature (18°C–25°C).

### Microscope set-up

To sedate worms and prepare the sample (i.e., to put a worm in the chip) we use a stereomicroscope, for example Nikon SMZ1000.

The microfluidics set-up (Figure 1V) consists of a pressure controller (Fluigent MFCS™-EZ) with 4 pressure outlets. Four 3 mL syringes are filled with the appropriate solutions (syringe 1 and 4 contain 5 μm fluorescein, syringe 2 contains your stimulus of interest and syringe 3 contains M13 buffer). The plungers of the syringes are punched with a custom-made steel pin, so that the pressure outlets can, via Tygon tubing, be connected to the syringes. The outlets of the syringes contain a valve, followed by Tygon tubing (1.6 mm ID × 3.2 mm OD). Via stainless steel pins of 0.025″ OD × 0.013″ ID the Tygon tubing is inserted in the microfluidics chip. We placed the syringes lower than the microscope stage where the device is placed, to prevent spontaneously flow due to gravity.

For fluorescence imaging we use a custom-built epi-illuminated wide-field fluorescence set-up.^3^^,^^4^ For flow calibration we use a 10× air objective (Nikon, Plan Fluor 10×, N.A.: 0.30). For calcium imaging (in the somas of the phasmid chemosensory neurons) we use a 40× air objective (Nikon, Plan Apo air 40×, N.A.: 0.95). For imaging protein dynamics inside the cilia we use a 100× oil immersion objective (Nikon, CFI Apo TIRF 100×, N.A.: 1.49) with an additional 1.5× magnification (inside the microscope body).

***Note:*** there are many alternative microscope set-ups possible, depending on what you have available in your lab and what your particular experiments require. Key is that it should be an inverted microscope, since it is not possible to image through the PDMS chip. We use a custom-built epi-illuminated wide-field system, but commercial wide-field systems should work as well. The same holds for other fluorescence microscopy modalities including point-scan confocal, line-scan confocal or spinning disk confocal. It is very useful to have access to bright-field imaging (in addition to fluorescence) on the same system, for example via the eyepiece. Other important considerations are the field-of-view and magnification of the system. When imaging of a substantial section of flow cell or worm is needed (e.g., imaging the whole tail of the worm in our calcium imaging experiments) a low-magnification (<40×) objective and/or a large area detector (e.g., a modern sCMOS camera) is needed. When only a small section of the worm is of interest (e.g., cilia, in our experiments), higher magnification, higher NA will be beneficial for high-resolution and high-sensitivity imaging. This is especially crucial when single-molecule sensitivity is required.

## Key resources table

**Table:** 

REAGENT or RESOURCE	SOURCE	IDENTIFIER
**Chemicals, peptides, and recombinant proteins**		

Sylgard 184 Polydimethylsiloxane (PDMS)	Dow Corning	N/A
Levamisole hydrochloride	Sigma-Aldrich	CAS 16595-80-5
Fluorescein		CAS 2321-07-5
Tris	Acros Organics	CAS 77-86-1
NaCl	Sigma-Aldrich	CAS 7647-14-5
KCl	Sigma-Aldrich	CAS 7447-40-7

**Experimental models: Organisms/strains**		

*C. elegans*	Caenorhabditis Genetics Center	N/A
*C. elegans*	Peterman Lab	N/A

**Other**		

Biopsy punches 0.75 mm	World Precision Instruments	504529
Plasma cleaner	Harrick Plasma	
Stainless steel pins (0.025″ OD × 0.013″ ID)	New England Small Tube corporation	NE-1310-06
3 mL syringes	Terumo	SS+03L1
Tygon Formula 2375 laboratory tubing (1.6 mm ID × 3.2 mm OD)	Merck Life Science	Z685585
Pressure controller	Fluigent MFCS™-EZ	MFCS-EZ-4

***Note:*** Use young adult worms for imaging. Select worms with a thickness that fits the worm trap of the microfluidics chip (see sample preparation). Depending on your experimental question you choose the worm strain (genotype).

## Materials and equipment

**Table:** M13 buffer

Reagent	Final concentration	Amount
1 M TrisHCl pH 7	30 mM	15 mL
NaCl	100 mM	2.92 g
KCl	10 mM	0.37 g
ddH_2_O	N/A	485 mL
**Total**	**N/A**	**500 mL**

Autoclave for 20 min at 121°C before use. Store at room temperature (18°C–25°C).

## Step-by-step method details

### Sample preparation

**Timing: ∼30 min**

This step involves the sedation of worms and inserting them into the worm trap of the microfluidics chip.

1. Sedate worms.
  a. Select ∼10 (young) adult worms from the strain you would like to image.
  b. Put them in a droplet of 10 μL 5 mM levamisole. Cover them with a small petri dish to avoid evaporation of the droplet, which would make the worms dry out.
  c. Incubate the worms for ∼15 min, till they are fully sedated and you cannot see them moving anymore.

***Note:*** Whether a worm does or does not fit well in the worm trap, depends on the worm’s thickness. The older the worms, the thicker they are. By experience, you will figure out which worms fit your chip best.

**Figure 2:**
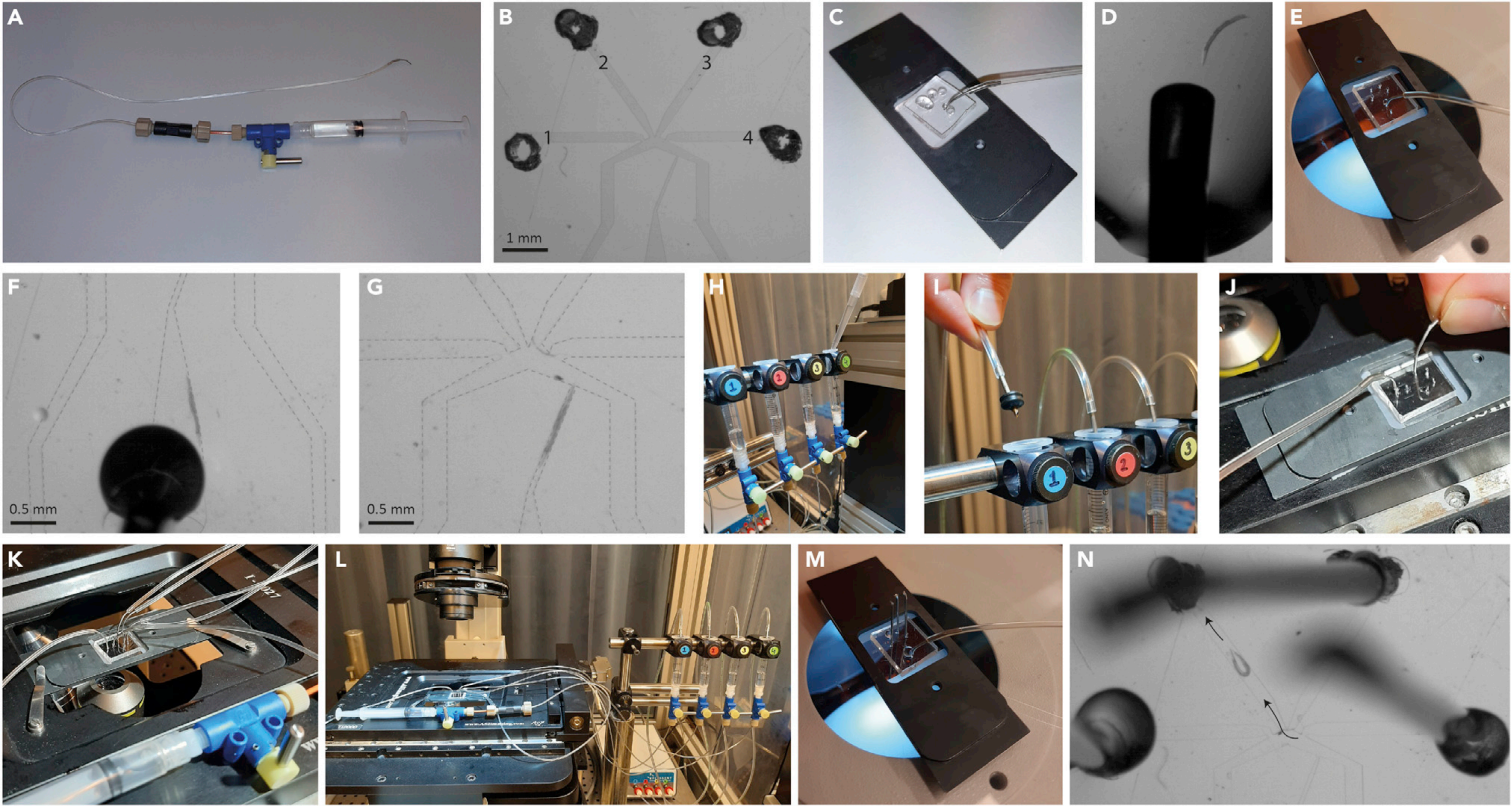
Sample preparation and microfluidics set-up (A) Stainless steel pin in Tygon tubing, which is via a valve connected to a 3 mL syringe filled with M13 buffer. (B) Picture of the microfluidics chip as presented by Chronis et al.^2^ Scalebar: 1 mm. The fluidic inlets are indicated by the number that corresponds to the syringe they have the be connected to. (C) Flush the chip with M13 buffer. (D) Suck up a worm head-first. (E) Insert the steel pin to the inlet of the chip’s worm channel to insert the worm. (F) Manually apply pressure to insert the worm. Dotted lines clarify the channels of the chip. Scalebar: 0.5 mm. (G) Worm in the chip’s worm trap, with the tail exposed to the fluidic streams. Dotted lines clarify the channels of the chip. Scalebar: 0.5 mm. (H) Fill four 3 mL syringes with the appropriate solutions: syringe 1 and 4 with 5 μm fluorescein; syringe 2 with your chemosensory stimulus and syringe 3 with M13 buffer. (I) Connect the syringes to a pressure controller via Tygon tubing. (J) Connect the syringes with Tygon tubing and a stainless-steel pin to the fluidic inlets of the microfluidics chip. (K) Put the sample under the microscope. (L) Microfluidics set-up with the sample under the microscope. (M) Close 3 of the 4 fluidic inlets with a closed steel pin, replace the steel pin that is connected to the M13-filled syringe to the waste outlet and manually apply pressure on the syringe to let the worm leave the chip via the (open) 4^th^ fluidic inlet. (N) Worm leaves the chip via channel 2. Arrow indicates direction of movement of the worm.

***Note:*** the Tygon tube is via a valve connected to a 3 mL syringe filled with M13 buffer.

***Note:*** this is an additional syringe, specifically used for worm loading, in addition to the four syringes that are connected to the pressure controller.

***Note:*** Ensure that all in- and outlets are open, i.e., not blocked, and do not contain air bubbles.

2. Use a stainless steel pin (New England small tube corporation, 0.025″ OD × 0.013″ ID) into a Tygon tube to flush the chip with buffer (Figures 2A–2C).

3. Carefully suck up a worm, head-first, using the stainless steel pin that is connected to the M13-filled syringe such that the worm is placed at the tip of the steel pin (Figures 2A and 2D).

***Note:*** We do this gently manually, with a not well-defined pressure.

4. Insert the steel pin to the inlet of the chip’s worm channel (Figure 2E) and manually apply pressure to the syringe to ensure the worm enters the worm trap (tail-first) (Figure 2F).
5. Gently keep applying pressure till the worm is at the appropriate position (Figure 2G).

***Note:*** If the worm does not enter smoothly or gets stuck halfway, it might help to change the direction of the flow back and forth slightly by pushing and pulling gently on the plunger.

6. Leave the syringe connected to the insert of the worm trap and close the valve. This will avoid the worm from being pushed back when you apply pressure on the fluidic inlets.

***Note:*** Sample preparation is a time-consuming step in the process. On a good day, we image 10–20 individual worms, which limits the throughput of this method.

### Calibration of microfluidic flows

**Timing: ∼15 min**

This step accomplishes the calibration of the microfluidic flows to ensure that the worm is only exposed to the chemosensory stimulus when the pressures are switched to ‘stimulus ON’ mode.

7. Fill four 3 mL syringes with the appropriate solutions: syringe 1 and 4 with 5 μm fluorescein; syringe 2 with your chemosensory stimulus and syringe 3 with M13 buffer (Figure 2H).
8. Connect the syringes to a pressure controller (Fluigent MFCS™-EZ) via Tygon tubing (Figure 2I).
9. Connect the syringes with Tygon tubing and a stainless-steel pin to the fluidic inlets of the microfluidics chip (Figure 2J).

***Note:*** The order in which you connect the syringes does not matter.

**Figure 3:**
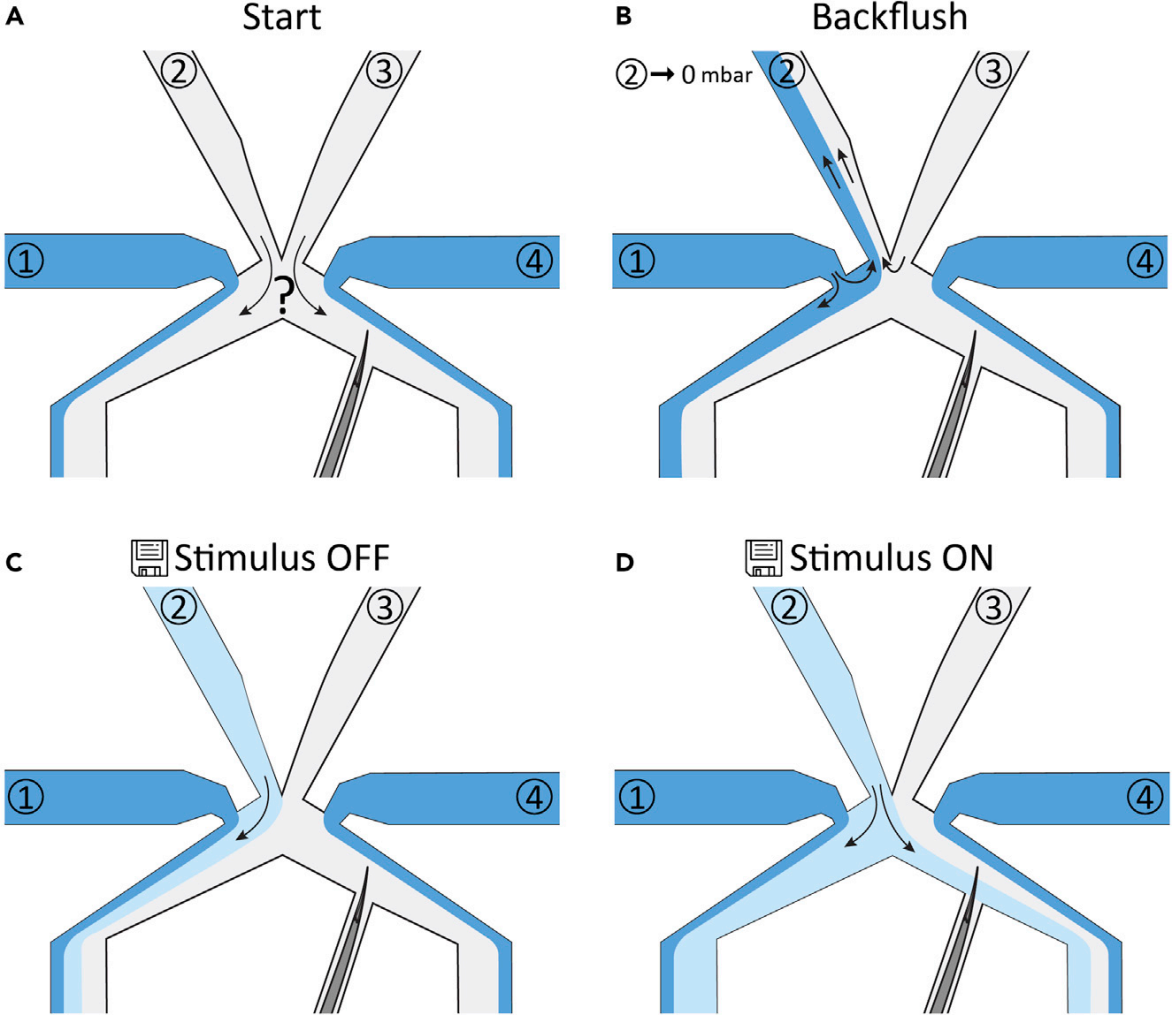
Calibration of microfluidic flows (A) Apply 100 mbar to channels 1, 3 and 4 and 90 mbar to channel 2. (B) Decrease the pressure on channel 2 to 0 mbar. A backflush of fluorescein from channel 1 and M13 buffer from channel 3 will occur. (C) Apply 100 mbar to channels 1, 3 and 4 and 90 mbar to channel 2. The lower concentration of fluorescein in channel 2 can now be distinguished from the fluorescein in channel 1 and 4 and the colorless buffer in channel 3. Determine the appropriate pressures for ‘stimulus OFF’ mode, so that fluid from channel 2 will not pass the worm’s tail. (D) Determine the appropriate pressures for ‘stimulus ON’ mode, so that fluid from channel 2 will pass the worm’s tail.
12. Gently increase and decrease the pressures on the different channels one by one to check if the flows are properly responding to changes in pressure.

***Note:*** If the flows do not respond properly to changes in pressure, there may be air bubbles in the tubing or the chip. Swiftly increase and decrease the pressure to let the air bubble get out of the way. See problem 2.

10. Put the sample under the microscope (a 10× objective works well) (Figures 2K and 2L).
11. Using the Fluigent All-in-one software (included with the pressure controller), apply 100 mbar to channels 1, 3 and 4 and 90 mbar to channel 2, to ensure no stimulus is delivered to the worm prior to starting the experiment (Figure 3A).

13. Decrease the pressure on channel 2 to 0 mbar. A backflush of fluorescein from channel 1 and M13 buffer from channel 3 will occur (Figure 3B). Wait for ∼10 s.
14. Put all pressures to 0 mbar, to allow mixing of the fluids. Wait for ∼10 s.
15. Apply 100 mbar to channels 1, 3 and 4 and 90 mbar to channel 2.

***Note:*** The lower concentration of fluorescein in channel 2 can now be distinguished from the fluorescein in channel 1 and 4 and the colorless buffer in channel 3 (Figures 3C and 3D).

16. Determine the appropriate pressures for ‘stimulus OFF’ and ‘stimulus ON’. Save the settings (Figures 3C and 3D).

***Note:*** The precise settings vary every time you do a new experiment, but typically we use pressures for Stimulus OFF: channel 1–25 mbar, channel 2–25 mbar, channel 3–35 mbar, channel 4–30 mbar; and for Stimulus ON: channel 1–30 mbar, channel 2–35 mbar, channel 3–30 mbar, channel 4–30 mbar.

***Note:*** You can now freely change the pressures in the different channels to find the right settings, because there is no stimulus coming from channel 2.

17. Switch to ‘stimulus OFF’ and wait till the fluorescein from channel 2 has disappeared.
18. Now the chemosensory stimulus is coming from channel 2 and you can start your measurement. Remain in ‘stimulus OFF’ mode for now.

### Imaging

**Timing: ∼10 min**

This step includes the actual measurement and data acquisition while exposing the worm to a chemosensory stimulus.

19. Depending on your research question, switch to an appropriate objective. We either use a 40× air objective or a 100× oil objective.
20. Bring the phasmid chemosensory cilia (or other region of your interest) in focus.
21. Start acquiring images for a certain time period.

***Note:*** This is your ‘baseline measurement’, when the worm is exposed to buffer and not yet to a chemosensory stimulus.

22. Switch pressures to ‘stimulus ON’. Write down frame number so that you remember when you switched the stimulus on. See problem 3.
23. When you want to stop the stimulus, switch back to ‘stimulus OFF’ mode. Again, write down the frame number.

***Note:*** The time frame to record the chemosensory response in *C. elegans* depends strongly on your scientific question. We typically expose the worm from several seconds to a few minutes. Performing repeated stimuli, prolonged exposure times or recovery experiments (in which you expose the worm to M13 buffer for a long time after exposure to a chemical stimulus) are all possible.

24. When you want to stop your measurement, stop acquiring images. Put all pressures to 0 mbar and disconnect the tubings from the chip.
25. Use the syringe that is still connected to the worm channel to push the worm out of the worm trap, by manually applying pressure to the syringe.
26. Close 3 of the 4 fluidic inlets with a closed steel pin, replace the steel pin that is connected to the M13-filled syringe to the waste outlet and manually apply pressure on the syringe to let the worm leave the chip via the (open) 4^th^ fluidic inlet (Figures 2M and 2N).
27. After the last experiment of the day make sure to clean the chip by flushing it with milliQ. The chip can be used multiple times, as long as it does not leak or is not clogged by dust or PDMS particles.

**Pause point:** Worms can be imaged for up to two hours after they have been sedated. Afterwards, it is advisable to sedate a new batch of worms.

## Expected outcomes

The protocol described here provides a reliable method to generate microfluidic chips and use those to expose (the tail of) *C. elegans* to chemosensory stimuli while recording the response by fluorescence microscopy. The expected outcome depends on the worm strain that you image. For examples of calcium imaging in the phasmid neurons or intraflagellar transport in the phasmid chemosensory cilia, we refer to our earlier work.^1^

## Limitations

The success of this protocol depends on the quality of the microfluidic chips. If the chips are properly immobilized on glass slides, they are quite robust to work with. If this is not the case and the chips are leaking, they cannot be used for experiments. The robustness varies from batch to batch. If you find that your chips are consistently leaking, it may help to increase the time of plasma cleaning.

In most cases, the chemosensory stimulus solutions cannot be distinguished from buffer under the microscope, since they are not fluorescent. Consequently, there is no positive control to verify that the worm actually got exposed to the stimulus. It is therefore very important to calibrate the flows very well. When the flows do not respond properly to changes in pressure, make sure to find and remove the cause (air bubbles and/or dust particles). In case the flows remain unreliable, you will have to change to a new chip.

Since this protocol involves sample preparation and imaging of only one worm at the time, throughput is relatively limited. Even though the worms are sedated, they often still move in response to aversive stimuli. Even the slightest movement out-of-focus or out of the field-of-view can be detrimental for imaging. This furthermore limits throughput. Patience and perseverance are essential!

## Troubleshooting

### Problem 1

Fluid is leaking where the steel pins are inserted in the chip. See before you begin - step 3c.

### Potential solution

Punching holes in the chip can cause small cracks in the PDMS. This will result in a poor seal between the chip and the steel pin and allows fluid to leak through. Often this happens when the puncher is blunt or damaged, therefore, replacing your biopsy punch will resolve this issue.

### Problem 2

The microfluidics chip leaks. See before you begin - steps 3f,g,h.

### Potential solution

This chip cannot be used for measurements and can be discarded. You will have to use another chip. If many chips within one batch leak, you should consider plasma cleaning the chips and glass slides for longer time.

### Problem 3

The flows do not respond properly to changes in pressure. See Method details - step 12.

### Potential solution

If the flows do not respond properly to changes in pressure, this can be explained by blockage of one (or several) of the channels, either by an air bubble or by a piece of PDMS or dust particle. Try to swiftly increase and decrease the pressure on the different channels to see if you can locate the problem. In most cases, the source of the problem will then have been flown away towards the waste channel. If this is not the case, you might need to disconnect the chip from the pressure box and flush the chip manually with M13 buffer.

### Problem 4

Worms move during stimulus exposure. See Method details - step 22.

### Potential solution

Although the worms are sedated and trapped in the chip, they can still exert movement in response to stimulus exposure. Movement (either in the z-direction or back in the worm trap) is detrimental for imaging. Picking worms of the right age (and thus thickness) will reduce movement significantly. If the worms are still moving, a freshly thawed vial of levamisole and/or a slightly longer incubation time might help.

## Resource availability

### Lead contact

Further information and requests for resources and reagents should be directed to and will be fulfilled by the lead contact, Erwin Peterman (e.j.g.peterman@vu.nl).

### Materials availability

The protocol described here did not generate new unique reagents. All *C. elegans* strains generated in Bruggeman et al.^1^ are available from the lead contact without restriction.

## Data Availability

Any additional information is available from the lead contact upon request.
